# Interrelated influence of light and Ni on *Trichodesmium* growth

**DOI:** 10.3389/fmicb.2013.00139

**Published:** 2013-05-29

**Authors:** Tung-Yuan Ho, Tse-Hua Chu, Cheng-Ling Hu

**Affiliations:** ^1^Research Center for Environmental ChangesAcademia Sinica, Taipei, Taiwan; ^2^Department of Earth Sciences, National Taiwan Normal UniversityTaipei, Taiwan

**Keywords:** *Trichodesmium*, light intensity, Ni, superoxide, superoxide dismutase

## Abstract

Our previous laboratory study revealed that insufficient Ni supply can limit nitrogen fixation in *Trichodesmium*, a primary diazotrophic phytoplankton in the tropical and subtropical oceans. Here we show that light intensity and Ni availability interrelate to influence *Trichodesmium* growth. *Trichodesmium* growth is severely inhibited under high light (670 μE m^–2^ s^–1^) and insufficient Ni condition. On the contrary, the sufficient supply of Ni in seawater can sustain the growth of *Trichodesmium* under either high or low light conditions. We also observed elevated intracellular Ni uptake in *Trichodesmium* grown under relatively high light condition, supporting that the Ni requirement is used for removing superoxide generated through photosynthetic electron transport. This study shows that light saturation condition for *Trichodesmium* growth is Ni concentration dependent. This finding may exhibit implications for interpreting temporal and spatial distributions and activities of *Trichodesmium* in both modern and ancient oceans when light intensity and Ni concentrations have significantly varied.

## INTRODUCTION

Biological pump is mainly driven by the supply of bioavailable nitrogen, a major limiting factor for phytoplankton growth in the ocean. Nitrogen fixation is a crucial process providing bioavailable nitrogen to marine ecosystem and thus plays an important role on influencing material cycling globally (Capone et al., 1997; Karl et al., 1997; Zehr and Kudela, 2011). Understanding how nitrogen fixation is regulated in the oceans may shed light on mechanisms controlling global carbon dioxide cycling and climate change (Falkowski, 1997; Sigman and Boyle, 2000; Canfield et al., 2010). One of the major diazotrophs in the tropical and subtropical ocean, *Trichodesmium*, has received intensive studies due to their dominant abundance and quantitative importance on nitrogen fixation in the ocean region (Davis and McGillicuddy, 2006; Westberry and Siegel, 2006). Previous studies show that the environmental factors, including temperature, light intensity, P and Fe concentrations, can be important parameters on influencing the growth of *Trichodesmium* (Capone et al., 1997; Karl et al., 1997; Berman-Frank et al., 2001; Sañudo-Wilhelmy et al., 2001; Mills et al., 2004). However, the major mechanisms for controlling the global distribution and activities of *Trichodesmium* are still not fully understood (Hood et al., 2004). *Trichodesmium* contains gene encoding Ni-containing superoxide dismutase (SOD; Palenik et al., 2003; Dupont et al., 2008), indicating that Ni can be essential metal in the cyanobacterium for removing superoxides. As nitrogenase is known to be irreversibly inactivated by oxygen and reactive oxygen species (ROS; Gallon, 1981), Ni and Ni-SOD can be particularly important for *Trichodesmium*, a non-heterocystous diazotroph carrying out oxygen-producing photosynthesis and nitrogen fixation simultaneously. Our previous study demonstrates that insufficient Ni supply can limit nitrogen fixation in *Trichodesmium* in both natural and artificial seawater when the supply of P and Fe is sufficient (Ho, 2013). We also found that increasing Ni concentrations elevates cellular SOD activities and nitrogen fixation rates, suggesting that Ni-SOD may be involved in the protection of nitrogenase from ROS production during photosynthesis in *Trichodesmium* (Ho, 2013). Indeed, studies on other diazotrophs showed that cellular SOD levels were elevated when they carried out nitrogen fixation (Gallon, 1981; Puppo and Rigaud, 1986), supporting that SOD might be involved in protecting the overall process of nitrogen fixation from the inhibition of superoxides in the diazotrophs (Puppo and Rigaud, 1986).

It is well-known that ROS are inevitably generated by photosynthetic electron transport in photosystem II in plant, particularly under high light condition (Asada, 1999, 2006; Mittler et al., 2004; Latifi et al., 2009). *Trichodesmium* dwells in the surface water of tropical and subtropical regions where light intensity of photosynthetic active radiation (PAR) may reach hundreds to a couple of thousands μE m^–2^ s^–1^ in the surface water during day time period (Carpenter et al., 1993). Field and laboratory studies also observed that the growth of *Trichodesmium* was light intensity dependent (Carpenter et al., 1993; Breitbarth et al., 2008). The photosynthetic rates of *Trichodesmium* were proportional to light intensities up to 2,500 μE m^–2^ s^–1^. In theory, cellular ROS production and accumulation would induce SOD expression and simultaneously elevate cellular uptake on the SOD co-factor metals to maintain cellular reproduction and growth. Here, we hypothesize that *Trichodesmium* require additional Ni intracellularly to maintain normal reproduction and growth under elevated light intensity due to the requirement to over-express SOD. We carried out laboratory culture experiments by varying light intensities and Ni concentrations to investigate the interrelated influence of light and Ni on the growth of *Trichodesmium*. We also determined the SOD activities and intracellular trace metal quotas of *Trichodesmium* grown in the diverse treatments to evaluate our hypothesis.

## MATERIALS AND METHODS

*Trichodesmium erythraeum* IMS101 was purchased from the NCMA, Provasoli-Guillard National Center for Marine Algae and Microbiota, USA. Cultures of *Trichodesmium* were grown in trace metal-clean polycarbonate bottles in a temperature-controlled growth chamber at 26°C with illumination varying from 30 to 670 μE m^–2^ s^–1^ (PAR) under a 12:12 h light–dark cycle. Photon irradiances were created by using different distance from light source and were verified by measuring light penetration PAR into a seawater-filled polycarbonate culture bottle using a submersible radiometer (Biospherical Instruments Inc. Model: QSL 2100).

The *Trichodesmium* IMS-101 strain was maintained in trace metal-defined culture media prepared by modified YBCII culture medium without containing fixed nitrogen (Chen et al., 1996; Ho, 2013). To prepare a trace metal-defined culture medium, we remove trace metal impurities from the salt solution of the YBCII medium by passing the artificial seawater through quartz column filled with Chelex-100 (Biorad, CA, USA) chelating resins. The background concentrations of trace metals were determined (Ho, 2013). Medium sterilization, medium preparation, and trace metal control and manipulation for the trace metal-defined YBCII media generally follows the procedures for preparing trace metal-defined culture media (Ho, 2013).

**FIGURE 1:**
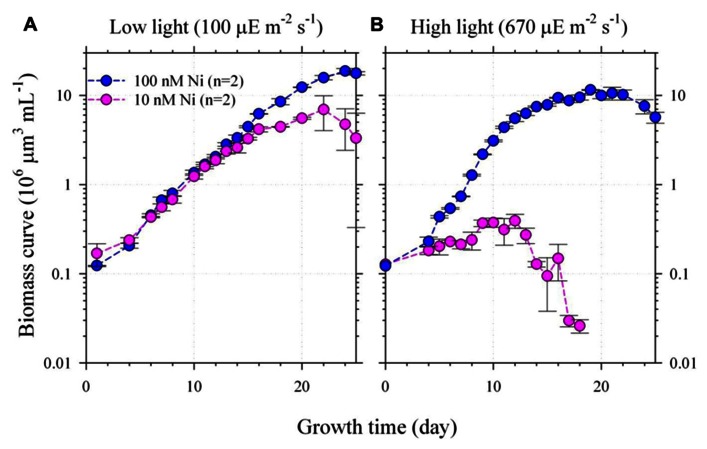
**The growth curves of *Trichodesmium* grown under the low (A) or high (B) light intensities (100 and 670 μE m^–2^ s^–1^) and low or high Ni concentrations (10 and 100 nM Ni)**. The inorganic Ni concentrations for 10 and 100 total dissolved Ni concentrations with 20 μM EDTA are equivalent to 6.7 and 67 pmol L^–1^ correspondingly (Westall et al., 1976). The biomass was presented by cellular volume measured by particle counter. The average deviation of the biomass measurement for duplicate bottles was generally less than 20%. The cellular SOD activities in the two-condition experiment were also determined during their late exponential growth period. Assuming the averaged cellular volume to be 250 μm^3^, the SOD activities measured during the late exponential growth period were 2.3 ± 0.2 μU per cell in the high light and high Ni treatment and the activities were only 1.1 ± 0.2 and 1.8 ± 0.2 μU per cell in the low light low Ni and low light high Ni treatments during the late exponential growth period, respectively. The biomass of the high light and low Ni treatment was too low to obtain the SOD activity.

**FIGURE 2:**
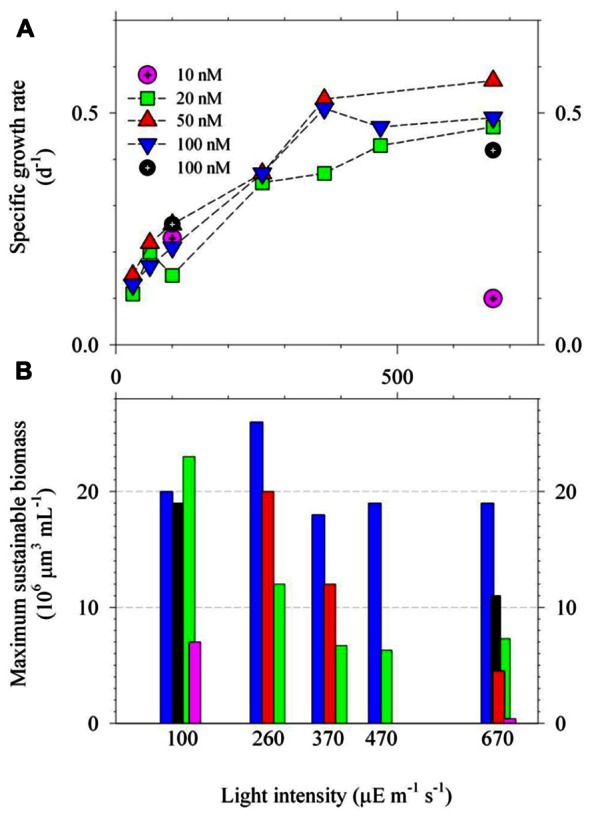
**The specific growth rates (A) and maximum sustainable biomass (B) of *Trichodesmium* grown under multiple-condition light and Ni treatments**. The light intensities ranged from 30 to 670 μE m^–2^ s^–1^ with intensities to be 30, 60, 100, 260, 370, 470, or 670 μE m^–2^ s^–1^ (PAR). The total dissolved Ni concentrations were 20, 50, or 100 nM, which are equivalent to 13, 33, and 67 pmol L^–1^ as inorganic concentrations, correspondingly. The specific growth rates were estimated by using data obtained from the exponential growth period. The *r*-square of linearity between growth time and the natural log of biomass were better than 0.98. The results of the growth rates of **Figure 1** are shown here by using black and pink circle symbols. Excluding the data of the treatments with 10 nM Ni, the growth rates in the low light treatments (<370 μE m^–2^ s^–1^) were significantly lower (*p* < 0.01) than the high light treatments (≥370 μE m^–2^ s^–1^). The biomass data for treatments with light intensities lower than 100 μE m^–2^ s^–1^ were not reported due to their low growth rates. The growth rate for the treatment with 470 μE m^–2^ s^–1^ and 50 nM Ni and the biomass data for the treatment with 100 μE m^–2^ s^–1^ and 50 nM Ni are not available due to operational issues.

Two culture experiments were designed and carried out in this study to test the hypothesis (**Figures 1** and **2**). The first experiment was designed to vary both light and Ni to two relatively extreme conditions (**Figure 1**). The light intensities and the total dissolved Ni concentrations were set to be 100 and 670 μE m^–2^ S^–1^ and 10 and 100 nM, respectively (**Figure 1**). The second experiment was to vary the light intensities and Ni concentrations to multiple conditions to further examine the relationship of the variations of the growth rates and intracellular trace metal quotas. The information of the light intensities and total dissolved and inorganic Ni concentrations ranged from 30 to 670 μE m^–2^ S^–1^ and from 20 to 100 nM, respectively. The details are shown in the **Figure 2**. Hereafter, we would mention the experiments in this study with the abbreviated terms, the two-condition or the multiple-condition experiments.

**FIGURE 3:**
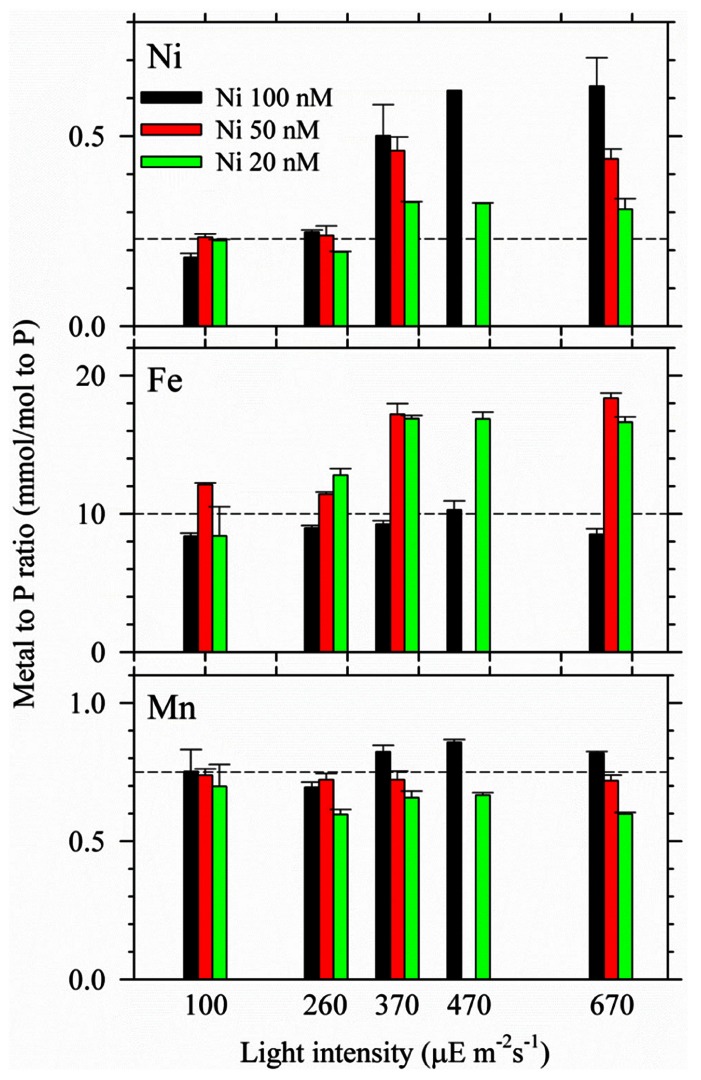
**Intracellular Ni, Fe, and Mn quotas in *Trichodesmium* grown under the multiple light and Ni conditions**. The trace metal quotas were all normalized to intracellular P concentrations. Cells were harvested during the late exponential phase. The error bar stands for analytical precision (one standard deviation of three replicate samples).

The ethylenediaminetetraacetic acid (EDTA) concentrations in the culture media for all treatments were 20 μM. The total dissolved Ni concentrations in the multiple-condition experiments were 20, 50, or 100 nM, which are estimated to be equivalent to 13, 33, and 67 pmol L^–1^ correspondingly as inorganic concentrations (Westall et al., 1976). The concentrations of phosphate and Fe were 50 μM and 500 nM for the two-condition experiment. To avoid precipitation and to accurately determine intracellular Fe quota (Ho et al., 2003), we decreased total dissolved Fe concentrations in the culture media to 100 nM (or 500 pM as inorganic form) for the multiple-condition experiment. Other trace metal concentrations in the medium were 20, 4, 1, 2.5, and 11 nM for Mn, Zn, Cu, Co, and Mo, respectively. The inorganic concentrations were 8.3 nM, 5 pM, 0.05 pM, and 5 pM for Mn, Zn, Cu, and Co, respectively (Westall et al., 1976). The two-condition experiment was carried out with duplicate bottles and the average deviation of the biomass for the duplicate bottles was generally less than 20% (**Figure 1**). The multiple-condition experiment included 21 different treatments so that we only used single bottle for the experiment due to the large sample number. The systematic trend observed for the growth curve and trace metal quotas indicates that the results obtained from single bottle were reliable (**Figures 2** and **3**). By using the average value obtained from the same light but different Ni treatments, the differences of the specific growth rates and the maximum sustainable biomass were statistically significant between the high light and relatively low light treatments. The detailed statistical results are shown in the **Figure 2**.

The biomass curves and the specific growth rates of *Trichodesmium* were determined by total cell volume per unit volume of seawater (μm^3^ mL^–1^), which was validated in previous studies (Goebel et al., 2008; Ho, 2013). The accuracy of the cell volume determined by particle counter was also validated by microscope (Ho, 2013). The SOD activities were determined using a cytochrome c with xanthine-xanthine oxidase method based on the ability of SOD to inhibit the reduction of cytochrome c by scavenging superoxide anion radicals produced by the xanthine-xanthine oxidase system (Flohe and Otting, 1984). One unit of SOD activity is defined as the amount of enzyme that produced a 50% inhibition of cytochrome c reduction. The calibration curve, with an *r*^2^ of more than 0.99 (*p* < 0.01), was first established between the reciprocal of the absorbance of ferrous cytochrome c and the enzymatic activity by using seven SOD standards. For each bottle, a linear correlation was observed between biomass and SOD activities. The SOD activity of each sample was obtained using the SOD assay for four different sample volumes. We then collected *Trichodesmium* samples through filtration, resuspended the cells in 1 mL seawater and determined the cell volume. We broke the cells using sonication under an ice-water slurry. After observing pigment release, we centrifuged the samples and removed the particulate material. Then, 100 μL of cell sample was added into a 3 mL quartz cuvette prefilled with 2.89 mL phosphate buffer solution, which contained xanthine and cytochrome C. Then, 10 μL xanthine oxidase was added into the cuvette for analysis (Flohe and Otting, 1984).

To determine intracellular trace metal quotas, cultured cells were harvested onto acid-washed 25-mm polycarbonate filters with 10 μm pore size in class 100 trace metal-clean laboratory. The filtered cells were rinsed with trace metal-clean seawater to remove the culture medium residue. The trace metal-clean seawater is the seawater passed through column filled with Chelex 100 chelating resins (Ho, 2013). Trace metal quotas in the phytoplankton were then determined using a HR-ICPMS (Thermo Scientific Element XR) fitted with a de-solvation system (Elemental Scientific). The details of the analytical precision, accuracy, and detection limits of the ICPMS (inductively coupled plasma mass spectrometry) method for seawater and phytoplankton were described in our previous studies (Ho et al., 2003, 2007).

## RESULTS

In the two-condition experiment, *Trichodesmium* were grown under 100 or 670 μE m^–2^ s^–1^ and in the culture media with 10 or 100 nM total dissolved Ni. The specific growth rates for the treatments under the low light–high Ni and the low light–low Ni conditions were similar, 0.26 and 0.23 d^–1^(**Figure 1A**), respectively. The maximum sustainable biomass of low light and high Ni treatment reached 20 × 10^6^ μm^3^ mL^–1^ (**Figure 1A**). However, under the high light–low Ni treatment (**Figure 1B**), the growth of *Trichodesmium* was significantly inhibited after slow growth for 10 days (*p* < 0.05). We have mainly used single-tailed and un-equal variances Student’s *t* test to examine if two samples are significantly different from each other in this study. The biomass only slowly increased from the initial biomass 0.1–0.5 (×10^6^ μm^3^ mL^–1^) in the first 10 days with specific growth rate (μ) to be 0.10 d^–1^ then the non-heterocystous diazotroph collapsed (**Figure 1B**). However, under the high light–high Ni condition (**Figure 1B**), *Trichodesmium* grew normally and the biomass exponentially increased from 0.1 to 2.0 (×10^6^ μm^3^ mL^–1^) in 10 days with specific growth rate up to 0.42 d^–1^ then gradually increased to 10 × 10^6^ μm^3^ mL^–1^. Under the low light–low Ni treatment (**Figure 1A**), the growth rate was similar to the low light–high Ni treatment and its biomass reached 7 × 10^6^ μm^3^ mL^–1^ (**Figure 1A**), which was 14-fold of the biomass for the high light–low Ni treatment (**Figure 1**). These results show that sufficient Ni supply is required for *Trichodesmium* to efficiently grow and reproduce under the high light condition.

We further carried out light and Ni interrelated experiment by using multiple light intensities and Ni concentrations. The results of their specific growth rates and the maximum sustainable biomass were presented in **Figure 2**. The variations of intracellular trace metal quotas of Ni, Fe, and Mn were also determined (**Figure 3**). Overall, **Figure 2** shows that the growth rates of *Trichodesmium* were both light intensity and Ni availability dependent (**Figure 2A**). Independent to Ni concentrations, the growth rates increased from 0.10 to 0.35 d^–1^ with increasing light intensities from 30 to 370 μE m^–1^ s^–1^ (**Figure 2A**). The growth rates mainly ranged from 0.42 to 0.57 d^–1^ for the treatments with light intensity higher than 370 μE m^–2^ s^–1^. With Ni concentrations higher than 20 nM, the specific growth rates under high light condition (≥370 μE m^–2^ s^–1^) were generally high and comparable.

## DISCUSSIONS

The results of **Figures 1** and **2** show that sufficient Ni supply is essential for *Trichodesmium* to grow efficiently under high light condition. With total dissolved Ni to be 100 nM or Ni′ to be 67 pM, the growth of *Trichodesmium* was light saturated at 370 μE m^–2^ s^–1^ and the growth rates reached 0.5 d^–1^ (**Figure 2**). The light saturated intensity and the corresponding growth rates observed in this study are both higher than previous studies reported for the same *Trichodesmium* strain. For example, Breitbarth et al. (2008) observed that the light saturated intensity of the species to be 180 μE m^–2^ s^–1^ with μ to be 0.26 d^–1^. It should be noted that most of previous culture studies commonly grew *Trichodesmium* under relatively low light condition, 90 or 100 μE m^–2^ s^–1^, by following the growth condition used in the study of Chen et al. (1996). These light intensities used in the culture studies are much lower than the light intensities *Trichodesmium* generally encounters in the surface water of the tropical and subtropical oceans and may result in low growth rates. We show that increasing Ni concentrations and light intensity would obtain elevated growth rates for *Trichodesmium* culture in laboratory. This finding also suggests that *Trichodesmium* blooming in the ocean may require Ni supply under high light condition.

Superoxide dismutase activities were measured in the two-condition experiments. Assuming the averaged cellular volume to be 250 μm^3^, the SOD activities measured during the late exponential growth period were 2.3 ± 0.2 μU per cell in the high light–high Ni treatment and the activities were only 1.1 ± 0.2 and 1.8 ± 0.2 μU per cell in the low light–low Ni and low light–high Ni treatments during the late exponential growth period, respectively. The biomass of the high light and low Ni treatment was too low to obtain the SOD activity. The SOD activities we obtained in the experiments were consistent to our previous study (Ho, 2013), in which the SOD activities and nitrogen fixation rates were determined under various Ni concentrations. The elevated SOD activities with elevated Ni availability validated the important role of Ni on the SOD expression in *Trichodesmium*.

In terms of the maximum sustainable biomass, the biomass ranged from 4 × 10^6^ to 26 × 10^6^ μm^3^ mL^–1^ for the treatments with Ni concentration over 20 nM (**Figure 2B**). With sufficient Ni supply, the maximum sustainable biomass seems to be independent to light intensities in the multiple-condition experiment (**Figure 2B**). In terms of Ni availability, 10 nM dissolved Ni concentrations (Ni′ = 6.7 pM) appears to be insufficient to maintain the growth rates and to sustain the biomass for all of the light treatments. Under light intensity to be 670 μE m^–2^ s^–1^, *Trichodesmium* barely grew in the medium with 10 nM Ni and the biomass only reached 4 × 10^6^ μm^3^ mL^–1^ in medium with 20 nM Ni. However, under 100 μE m^–2^ s^–1^, the biomass of the treatments with 10 and 20 nM Ni were much higher than the high light treatment, reaching 7 × 10^6^ to 23 × 10^6^ μm^3^ mL^–1^, respectively. For relatively high light treatments, the total sustainable biomass for the treatments with 20 nM Ni was generally lower than the treatments with 100 nM Ni. For treatments with 20 and 50 nM Ni, the maximum cellular volumes at 670 μE m^–2^ s^–1^ were only 4.5 and 7.3 μm^3^ mL^–1^, possibly showing growth stress induced by the combined effect of strong light and the lack of bioavailable Ni. Although the 20 and 50 nM total Ni concentrations may be high enough for *Trichodesmium* to grow normally at the beginning stage the bioavailable concentrations may not be high enough to sustain the growth rates at the later period with relatively high biomass (**Figure 2**). Our previous study has demonstrated that nitrogen fixation rates in *Trichodesmium* are Ni availability dependent (Ho, 2013). The inconsistency between the growth rates and the maximum sustainable biomass may be attributed to the influences of Ni availability on its nitrogen fixation rates. Overall, the results of **Figures 1** and **2** indicate that the growth of *Trichodesmium* under relatively high intensity is Ni availability dependent and light saturating or inhibiting condition in *Trichodesmium* is Ni concentration dependent. When Ni supply is sufficient in culture medium (Ni > 20 nM or Ni′ > 13.4 pM), the specific growth rate of *Trichodesmium* can be up to 0.5 d^–1^ at light intensity higher than 370 μE m^–2^ s^–1^.

Intracellular trace metal quotas were presented by normalizing the cellular concentrations to phosphorus (**Table 1; Figure 3**). It should be noted that *Trichodesmium* also contains gene encoding Mn-containing SOD (Dupont et al., 2008). However, no significant evidence has been found for the mutual replacement between Ni and Mn in *Trichodesmium*. We did not observe considerable Mn quota changes in *Trichodesmium* grown in the media varying Ni concentrations from 10 to 200 nM in our previous study either (Ho, 2013), suggesting that Mn-SOD may not be involved in removing the superoxide generated in chloroplasts. Similarly, the Mn quota did not significantly change among different light intensities and Ni concentrations in this study (**Figure 3**). The *p* value of the *t* test for the Mn quota between any two light intensities are all larger than 0.2. Indeed, the Mn quota seemed to slightly increase with increasing Ni concentrations at the same light intensity for the high light treatments (≥370). Both Ni and Fe quotas varied significantly with light intensities and Ni availability. The Ni quota for the cells grown under the high light treatments (≥370) were significantly higher than the quota of the cells grown in the treatments with lighter intensity lower than 370 μE m^–1^ s^–1^ (*p* < 0.05). The Ni quota increased from 0.18 to 0.62 mmol/mol P with increasing light intensity for the 100 nM treatments; increased from 0.23 to 0.45 mmol/mol P for the 50 nM treatment; and only increased from 0.23 to 0.30 mmol/mol P in the treatment of 20 nM Ni. The increasing trend of the Ni quota indicates that intracellular Ni requirement was elevated in the cells with increasing light intensity or ROS production. Both treatments with 20 and 50 nM Ni in the media probably were not high enough to provide *Trichodesmium* sufficient Ni to form Ni-SOD for removing superoxide, which is consistent with the relatively low biomass observed for the high light treatments (≥370) shown in **Figure 2B**. In terms of Fe quota, although total Fe concentration was lowered down to 100 nM in the culture medium, we did not see significant change of Fe quotas with varying light intensity when Ni was 100 nM (*p* = 0.11), suggesting that the 100 nM Fe may be adequate for *Trichodesmium* at high light and 100 nM Ni condition. For the treatments with 20 and 50 nM Ni, the cellular Fe quotas were significantly higher (*p* < 0.05) for the high light treatments (≥370) than the relatively low light treatments (<370). We assume that the variations of Fe quotas with light intensity and Ni availability may be related to the associated interaction with Fe catalases or uptake hydrogenases. For example, the accumulation of the superoxide due to insufficient Ni-SOD activity may enhance the over-expression of catalases and elevate Fe uptake or intracellular Fe quota.

**Table 1:** **Trace metal quotas to P (mmol/mol) in *Trichodesmium* grown under various Ni availability and light intensity (μE m^–2^ s^–1^)**. The total dissolved and the estimated inorganic Ni (Ni’) concentrations in the culture media are shown in the first column.

Ni (Ni’) (nM/pM)	Light intensity	Mn/P	Fe/P	Ni/P
20 (13)	100	0.70 ± 0.08	8.4 ± 2.1	0.23 ± 0.01
	260	0.60 ± 0.02	12.8 ± 0.5	0.20 ± 0.01
	370	0.66 ± 0.02	16.9 ± 0.2	0.33 ± 0.01
	470	0.67 ± 0.01	16.9 ± 0.5	0.32 ± 0.01
	670	0.60 ± 0.01	16.6 ± 0.4	0.31 ± 0.03
50 (33)	100	0.74 ± 0.02	12.1 ± 0.1	0.23 ± 0.01
	260	0.72 ± 0.02	11.4 ± 0.1	0.24 ± 0.02
	370	0.72 ± 0.03	17.2 ± 0.8	0.46 ± 0.04
	670	0.72 ± 0.02	18.4 ± 0.4	0.44 ± 0.03
100 (67)	100	0.75 ± 0.03	8.4 ± 0.2	0.18 ± 0.01
	260	0.70 ± 0.02	9.0 ± 0.2	0.25 ± 0.01
	370	0.82 ± 0.01	9.3 ± 0.2	0.50 ± 0.08
	470	0.86 ± 0.06	10.3 ± 0.6	0.62 ± na
	670	0.82 ± 0.03	8.5 ± 0.4	0.63 ± 0.07

In conclusion, the data of this study show that light intensity and Ni availability interrelate with each other in controlling the growth and activities of *Trichodesmium*. We have demonstrated that *Trichodesmium* requires sufficient Ni to maintain elevated reproduction and growth rates under elevated light intensity conditions. The condition of photo-saturation for *Trichodesmium* growth is Ni concentrations dependent. As light intensity varies dramatically temporally and spatially in oceanic surface water of the tropical and subtropical region our finding might exhibit major implications for modeling temporal and spatial distributions and activities of *Trichodesmium* in both modern and ancient oceans when light intensity and Ni concentrations have changed.

## Conflict of Interest Statement

The authors declare that the research was conducted in the absence of any commercial or financial relationships that could be construed as a potential conflict of interest.
